# Biobased and Programmable Electroadhesive Metasurfaces

**DOI:** 10.1021/acsami.2c10392

**Published:** 2022-10-06

**Authors:** Qinyu Li, Antoine Le Duigou, Jianglong Guo, Vijay Kumar Thakur, Jonathan Rossiter, Liwu Liu, Jinsong Leng, Fabrizio Scarpa

**Affiliations:** † Bristol Composites Institute, University of Bristol, BS8 1TRBristol, U.K.; ‡ Polymer and Composites, Université Bretagne Sud, IRDL UMR CNRS 6027, F-56100Lorient, France; § School of Science, Harbin Institute of Technology (Shenzhen), Shenzhen518055, P. R. China; ∥ Biorefining and Advanced Materials Research Center, Scotland’s Rural College (SRUC), Kings Buildings, West Mains Road, EH9 3JGEdinburgh, U.K.; ⊥ School of Engineering, University of Petroleum and Energy Studies (UPES), Dehradun248007, Uttarakhand, India; # SoftLab, Bristol Robotics Laboratory, University of Bristol, Ada Lovelace Building, University Walk, BS8 1TWBristol, U.K.; ∇ Department of Astronautical Science and Mechanics, Harbin Institute of Technology (HIT), P.O. Box 301, No. 92 West Dazhi Street, Harbin150001, P. R. China; ○ National Key Laboratory of Science and Technology on Advanced Composites in Special Environments, Harbin Institute of Technology (HIT), No. 2 Yikuang Street, P.O. Box 3011, Harbin150080, P. R. China

**Keywords:** metasurfaces, hygromorph, electroadhesive, natural fibers, shape morphing

## Abstract

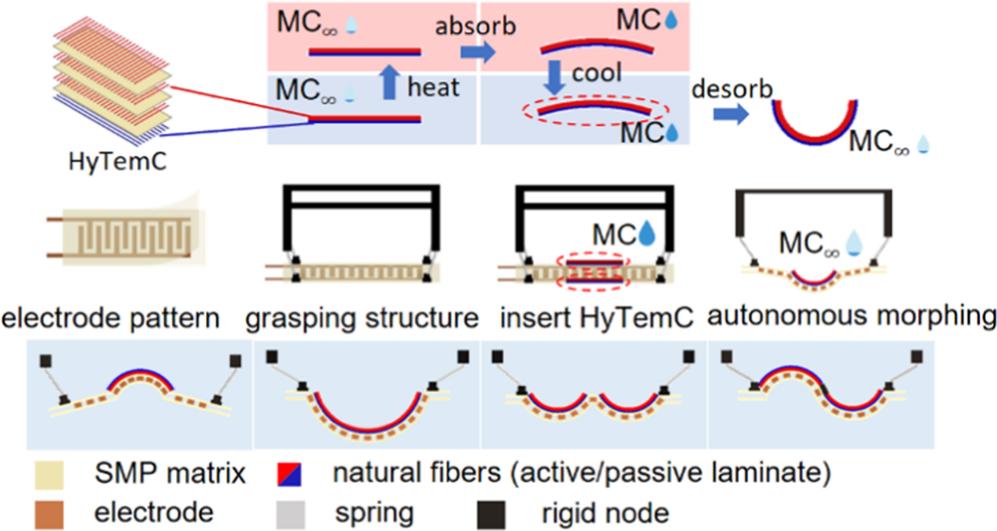

Electroadhesion has shown the potential to deliver versatile handling devices because of its simplicity of actuation and rapid response. Current electroadhesion systems have, however, significant difficulties in adapting to external objects with complex shapes. Here, a novel concept of metasurface is proposed by combining the use of natural fibers (flax) and shape memory epoxy polymers in a hygromorphic and thermally actuated composite (HyTemC). The biobased material composite can be used to manipulate adhesive surfaces with high precision and controlled environmental actuation. The HyTemC concept is preprogrammed to store controllable moisture and autonomous desorption when exposed to the operational environment, and can reach predesigned bending curvatures up to 31.9 m^–1^ for concave and 29.6 m^–1^ for convex shapes. The actuated adhesive surface shapes are generated via the architected metasurface structure, incorporating an electroadhesive component integrated with the programmable biobased materials. This biobased metasurface stimulated by the external environment provides a large taxonomy of shapes—from flat, circular, single/double concave, and wavy, to piecewise, polynomial, trigonometric, and airfoil configurations. The objects handled by the biobased metasurface can be fragile because of the high conformal matching between contacting surfaces and the absence of compressive adhesion. These natural fiber-based and environmentally friendly electroadhesive metasurfaces can significantly improve the design of programmable object handling technologies, and also provide a sustainable route to lower the carbon and emission footprint of smart structures and robotics.

## 1 Introduction

Electroadhesion (EA) is the electrostatic effect that generates bonding between two contacting surfaces subjected to a controllable electrical field.^1,2^ EA technologies have been attracting wide interest in recent years because of several beneficial aspects. Electroadhesion can be applied over many different substrates (including paper, glass, and metal)^3^ and can be operated in various working environments—from dusty terrestrial to low-pressure outer space.^4^ Electroadhesive systems are simple to operate and do not require energy-intensive pumps or control-intensive electric motors.^5^ EA technologies are characterized by low energy consumption (i.e., currents on the order of μA)^3,4^ and are adapted to handle and lift delicate objects through contactless or soft contacting pads.^6,7^ Electroadhesion has been used to deliver a range of end effectors for gripping, manipulation, and assembly tasks.^3,8−14^ Current EA grippers can be classified into three different types according to their adhesive surfaces: rigid,^8^ flexible or compliant,^3,10−12^ and stretchable.^9,13,14^ Examples of compliant EA grippers are represented by layers of elastic foam^11^ or semiflexible mountings between the gripping surface and the main substrate;^10^ the latter offer enhanced adaptation to the surfaces of the rigid objects, from flat to nonflat (concave and convex) exteriors. To fit a variety of object surfaces (flat, curved, and irregular), EA grippers have been designed to change shape using controllable motors,^12^ pneumatic pumps (PneuEA gripper),^14^ and dielectric elastomer actuators (DEA-EA soft gripper^9^ and EA-DEA soft composite gripper).^13^ These shape adaptive EA grippers, however, cannot grasp flat, highly curvy surfaces and even wavy-shaped and extremely fragile objects without the application of external forces or the use of heavy devices to control the adhesive surfaces.

In contrast to traditional heavy and high energy consumption mechanical motors, smart stimuli-responsive materials provide an appealing option to develop adhesive surfaces that are changeable and adaptive. Hygromorph natural fiber composites,^15,16^ with bilayer architectures, are inspired from hydraulic actuators present in nature, such as pinecone scales. Hygromorphs can generate remarkable actuation stresses (up to around 100 MPa^17^) that are significantly higher than those of classical smart materials including shape memory polymers (SMPs),^18^ electroactive polymers,^19^ hydrogels,^20^ and liquid crystal elastomers.^21^ Hygromorph composites are stimulated by humidity gradients available in the surrounding environment. In contrast, the high temperatures,^18,20,21^ and electric fields,^19^ used to stimulate other classes of smart materials typically rely on external devices such as electric heaters or electromagnetic coils. In addition, hygromorph composites made of natural fibers, such as flax and hemp, are sustainable and environmentally friendly. Limitations of the currently available natural fiber hygromorphs are related to their one-to-one relationship between changing shapes and humidity conditions, which implies the use of a single and constant actuated shape in the operational environment. The programmable and reconfigurable hygromorph composites presented here combine natural fibers and shape memory polymer matrices to overcome the one-to-one limitation. This new class of sustainable smart material actuators allows various predefined and actuated shapes to emerge in different working environments by implementing different programming steps.^22^ Hygromorph composites with intrinsic material programming features constitute a potential class of actuators that generate adhesive characteristics to match different surfaces of objects. Quite importantly, the use of flax fibers in these biobased composites provides a sustainable route to develop smart adaptive solids with load-bearing capacity^23^ and low carbon footprint.^24^

Flexible metasurfaces have been designed to manipulate the performance of terahertz and optical metamaterials by bending, stretching, and rolling flexible substrates.^25−27^ Examples range from thin conductive strips on flexible high permittivity pad^28^ to indium tin oxide (ITO)-coated poly(ethylene terephthalate) (PET) films on poly(vinyl chloride) (PVC) substrates.^29^ Metasurfaces based on EM modulation can be made self-reconfigurable,^30^ patterned for surface-enhanced Raman spectroscopy,^31^ and provide biomolecular sensing at the interface between chiral and hyperbolic metamaterials.^32^ One of the advantages of flexible metasurfaces is the miniaturization of the “hard” metamaterial cells because of the shape adaptability offered by ultrathin architected metallic substrates.^33^ Metasurfaces are a critical design paradigm to harness functionalities in nonlinear optical metamaterials.^34^ The propagation of mechanical and acoustic waves can also be tailored using patterned and elastic substrates.^35−38^ Shape memory polymer (SMP) substrates have also been used to develop self-deformable spatial modulation metasurfaces for realizing electromagnetic beam splitting and steering capabilities.^39^ Shape memory in alloy forms has also been recently used to develop multifunctional thermos-mechanical anisotropy.^40^ The flexible metasurface is therefore a promising design paradigm to add functionalities associated with localized and global shape change of substrates and to develop adaptive structures for robotics and object handling.

Here, we demonstrate a novel method to create ultracomplex shape electroadhesive systems based on environmentally driven morphing via a metasurface design. The metasurface conforms to the surface of objects using programmable hygrothermal biocomposite (HyTemC) actuators combined with surface electroadhesion. These biocomposites are architected around a structural configuration that provides the possibility of adapting to flat, highly curved (concave and convex), waved, and bimodal adhesive shapes. Adhesion is provided by the electroadhesion force, and the minimal compressive pressure exerted suits the handling of extremely fragile objects. The work here is organized as follows. The conceptual design and fabrication of the sustainable biocomposite gripper are described first. The design of the laminate HyTemC and the optimization of the electrode pattern for the electroadhesion is then illustrated. The material programming process of the biocomposite metasurface bonding with various geometrical objects is evaluated in different cases. Conclusions and future work are then presented in the final section.

## 2 Concept and Materials for the Biobased Electroadhesive Metasurface

### 2.1 Concept

**Figure 1:**
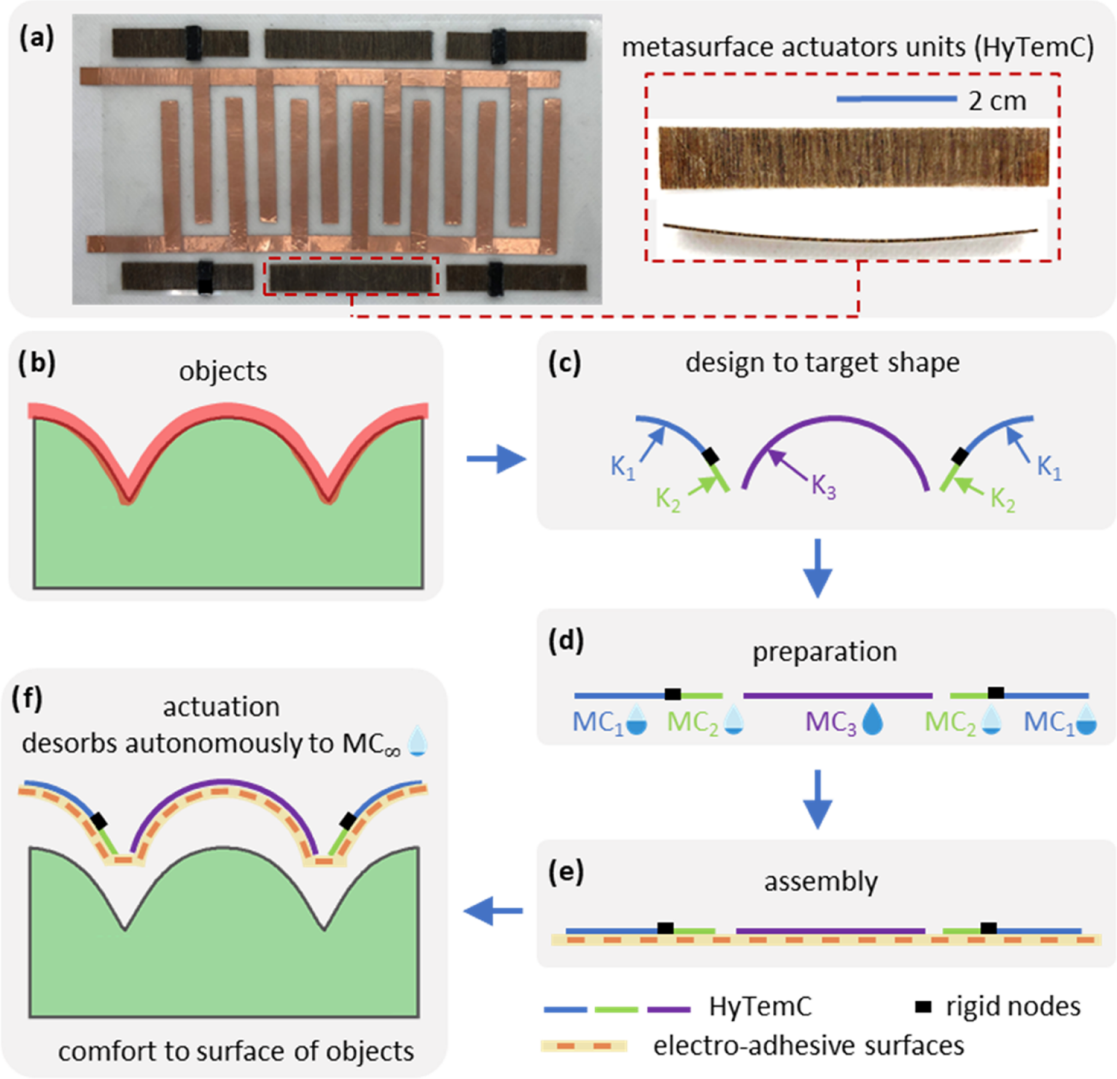
Demonstration of the biobased and programmable metasurface with electroadhesive capabilities (a). The electroadhesive surfaces of the objects (b) can be designed into several parts with different curvatures when considering the target surface to bond (c). The programmed biocomposite substrates are assembled to metasurfaces (d, e) with adhesive surfaces that conform to the same shapes as the target objects (f).

The actuator developed using biobased and electroadhesive adaptive metasurface is the combination of a flexible electroadhesive substrate embedded within an environmentally driven material to control the contacting surfaces. The environmentally driven metasurface is made of biobased hygromorph materials. The target complex adhesion surfaces are decomposed into a series of circular curves with different curvatures (*K*) and arc lengths (Figure 1). The targeted adhesive geometry is achieved by programming the metasurface with a set of initial moisture content (MC) parameters, which correspond to the bending curvatures, length, number of strips/units, and connectivity to the handled object (continuous or discontinuous). The programmed metasurfaces are initially assembled with compliant electroadhesive double-sided tapes and actuated to obtain the designed complex shapes. Table 1 shows a comparison of existing actuators available for electroadhesive grippers. Prior prototypes are based on pneumatics and dielectric elastomer actuators (DEAs). The existing actuators are integrated and allow for flat and bending shapes (all in one direction). On the contrary, the biobased metasurfaces presented in this work can create more complex bonding surfaces due to their multielement architecture. The multiple shapes and environmentally stimulated metasurface used in this work are based on the hygrothermal morphing biocomposites.^22^

**Table 1:** Comparison of Existing Actuators with Electroadhesive Surfaces

	adaptive shapes	variable stiffness	actuation method
DEA-EA soft gripper^9^	flat, sphere, cylinder,	no	electricity
EA-DEA soft composite gripper^13^	flat and concave	no	electricity
PneuEA gripper^14^	flat, sphere, cylinder	no	pneumatic
our work	flat, circular, single/double concave and wavy, piecewise, polynomial, trigonometric, and airfoil shapes	yes	autonomous desorption in the operational environment

**Figure 2:**
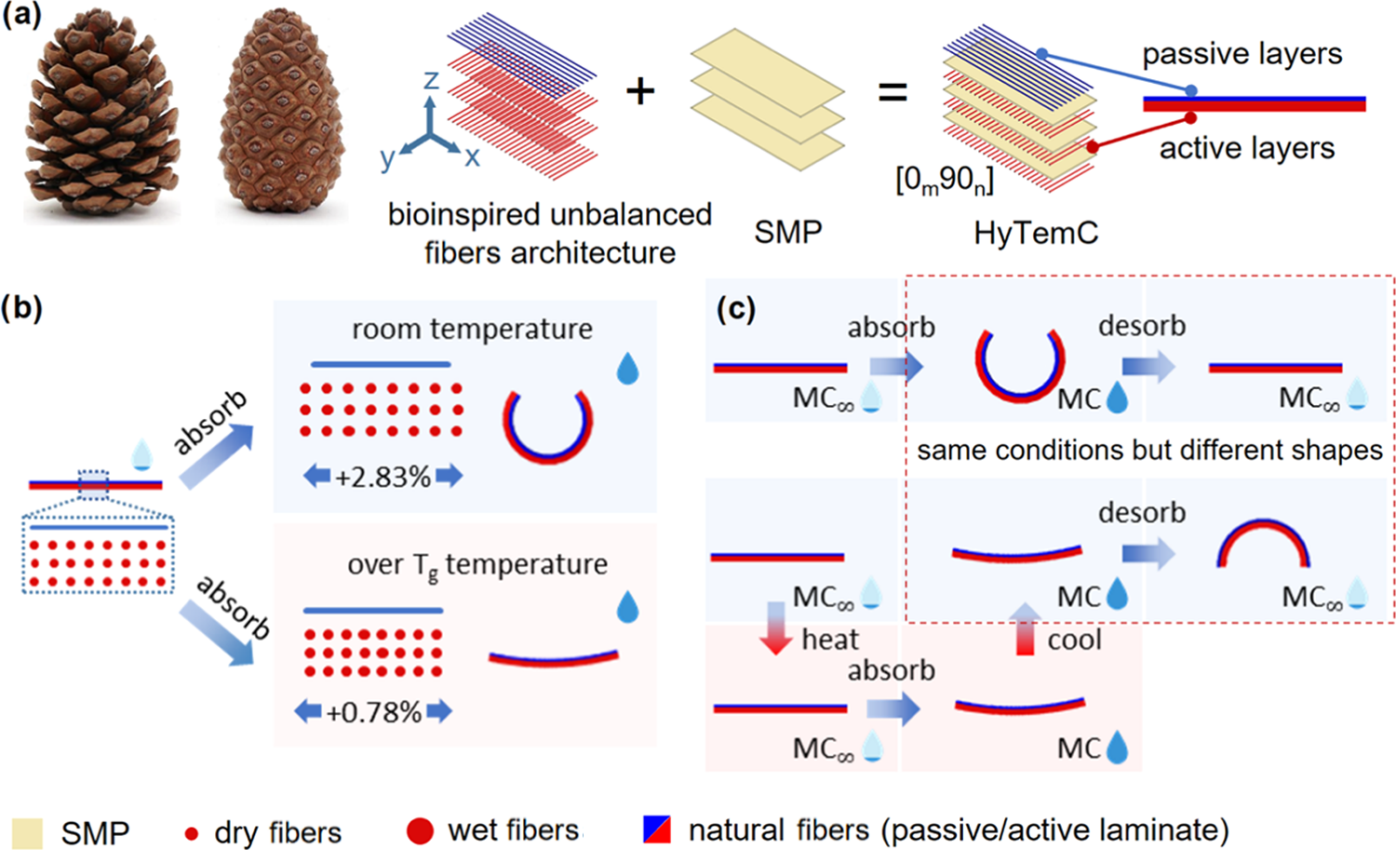
Concept and mechanism of the HyTemC as the biobased metasurface. (a) HyTemC is the combination of bioinspired unbalanced fibers architecture and SMP marked as [0*_m_*90*_n_*], meaning that the bilayer microstructure architecture has m laminas in the longitudinal direction as passive layers and *n* laminas along the transverse direction as active layers. (b) Induced temperature stimulus creates two different moisture absorptions at room temperature and over *T*_g_ temperature with totally different hygroscopic expansion of active layers, 2.83% and only 0.78% at room and over *T*_g_ temperature, respectively. Less swelling of active layers and the ability of SMP to keep original flat shapes act together to make the nearly flat shapes of HyTemC over *T*_g_ temperature, although absorbing a similar amount of moisture as the room temperature condition. (c) Heated HyTemC can absorb and store specific MC values but would maintain nearly flat shapes and then cool down as the predefined metasurface configuration. When exposed to an operational environment, the stored moisture will desorb autonomously, and the metasurface will reach the designed curvature. The square samples undergo the exact same environmental conditions but possess totally different shapes because of the temperature stimulus.

The HyTemC is formed from an unbalanced stack of natural fibers (flax) in a shape memory polymer (SMP) epoxy matrix (Figure 2a). The actuation principle is inspired by the closing and opening offered by the internal architecture of pinecone scales.^41^ The bioinspired fibers architecture consists of soft and high swelling ratio active layers and stiff and low hygroscopic expansion ratio passive laminas. The unbalanced microstructure of the composite material causes the bending of the composite stack due to the change of the moisture content, while the SMP matrix is sensitive to environmental temperature. Different from conventional single humidity stimulus hygromorph composites, the hygro-temperature coupled fields here create new types of bending shapes (Figure 2b). The curvatures of the HyTemCs change in different ways at room temperature and over *T*_g_, while these composites absorb the same amount of moisture. One reason for this behavior is the different hygroscopic expansion of the active layers (2.83% at room temperature) but only 0.78% over the *T*_g_ temperature. The other reason for the numerous moisture-induced curvature changes at different temperatures is the ability of the SMP matrix to resist bending motions to reach highly curved shapes. Nearly flat shapes of the composite materials occur over *T*_g_; the HyTemCs can therefore be heated over the *T*_g_ temperature to absorb and store specific moisture contents and cooled down to assume the preprogrammed shape. Those metasurfaces possess moisture contents (MCs) that will desorb autonomously when exposed to the working environment and will also morph into the expected shapes that correspond to the predefined curvatures at those specific MCs (Figure 2c). Via temperature, it is therefore possible to create several new sets of shapes at varying humidity conditions.

The selection of the HyTemC hygromorphs and biobased composite materials as actuators provides some significant advantages. The HyTemCs change shape without any external device generating the stimulus (such as an electrical or a pneumatic power source). Our autonomous metasurfaces maintain the operational simplicity of EA grippers. The integrated HyTemCs can be also cut into virtually any dimension. Elements made with hygromorph materials can therefore provide complex shapes with high resolutions and minimum size because of the intrinsic dual shape memory and hygroscopic strain capabilities at the microstructure material level. The HyTemCs also provide a large range of curvature deformations based on different microstructure laminate designs (see Section 3.1) and high actuation stresses (up to 88.6 MPa).^22^ The actuation process is based on the moisture desorption, with its tensile modulus passing from 6.6 GPa at immersed state to 13.8 GPa at 50% relative humidity (RH)—see Figure S1e,f. The adhesive surfaces are soft during the shape-changing process (i.e., the desorbing process) and then transition to rigid when actuated (desorbed). This prevents peeling, especially when the lifted objected have weights above a certain threshold.^42^

### 2.2 Details of the HyTemC Biobased Materials

The HyTemC composites consist of flax fibers and a thermoset shape memory polymer (SMP) matrix (Figure 2a). Unidirectional pure flax-fiber tapes (50 g/m^2^) have been supplied by Nat-up France. Flax fibers are fixed by tape on the edges of the samples and cut into 250 mm × 250 mm size by scissors. Stacks of SMP films provided by Leng’s group^43^ and unidirectional flax-fiber tapes have been cured in an autoclave at 0.69 MPa pressure, heated for 80 °C (3 h), 100 °C (3 h), and 150 °C (5 h). The unbalanced architecture across the thickness results in laminates with stacking sequences equal to [90_8_], [0_1_90_7_], [0_2_90_6_], and [0_3_90_5_] ([0*_m_*90*_n_*]), with *m* laminas in the longitudinal direction and *n* laminas along the transverse one. The cured composites have ∼0.56 mm total thickness, 40% fiber content, and 15.58 ± 1.51% porosity content,^22^ the latter being determined via gravimetric measurements in water.^44^ Other material properties critical for the actuation (mass diffusion, hygroscopic expansion, and mechanical tensile parameters along the longitudinal and transverse direction with varying temperature and moisture) are reported.^22^ The composite plates are cut into small stripes of 10 mm in width and variable length, based on the classes of objects to be grasped. Unbalanced laminates like [0_1_90_7_], [0_2_90_6_], and [0_3_90_5_] have passive layers in the longitudinal direction (0*_m_*) and active layers along the transverse one (90*_n_*). Passive layers are stiff with low swelling ratios, while active layers are soft with high hygroscopic expansion ratios. The morphing via bending occurs with the change of the environmental humidity that triggers different hygroscopic expansions between active and passive layers. Once manufactured, the unbalanced biobased composites have a one-to-one relationship between the environmental humidity and the actuated shapes. Again, different from conventional unbalanced natural fiber composites, the SMP matrix in our hygromorphs provides a programming capability to absorb and store specific controllable moisture levels over the matrix *T*_g_ and maintaining—at the same time—nearly flat shapes for the composites (Figure 2b). Swelling ratios of the active layers over the *T*_g_ decrease dramatically from those at room temperature.^22^ Once cooled, the nearly flat shapes with specific moisture contents (0–19.41% for the [0_2_90_6_] laminates) will desorb and morph autonomously to reach various actuated shapes. More moisture to desorb corresponds to larger deformations due to the actuation, which lead to a variety of actuated shapes that are continuously available. Those different shapes can be predesigned due to the controllable initial MC values during programming, even when the operational environmental humidity has a constant value.

### 2.3 Electroadhesive Metasurface Structure

**Figure 3:**
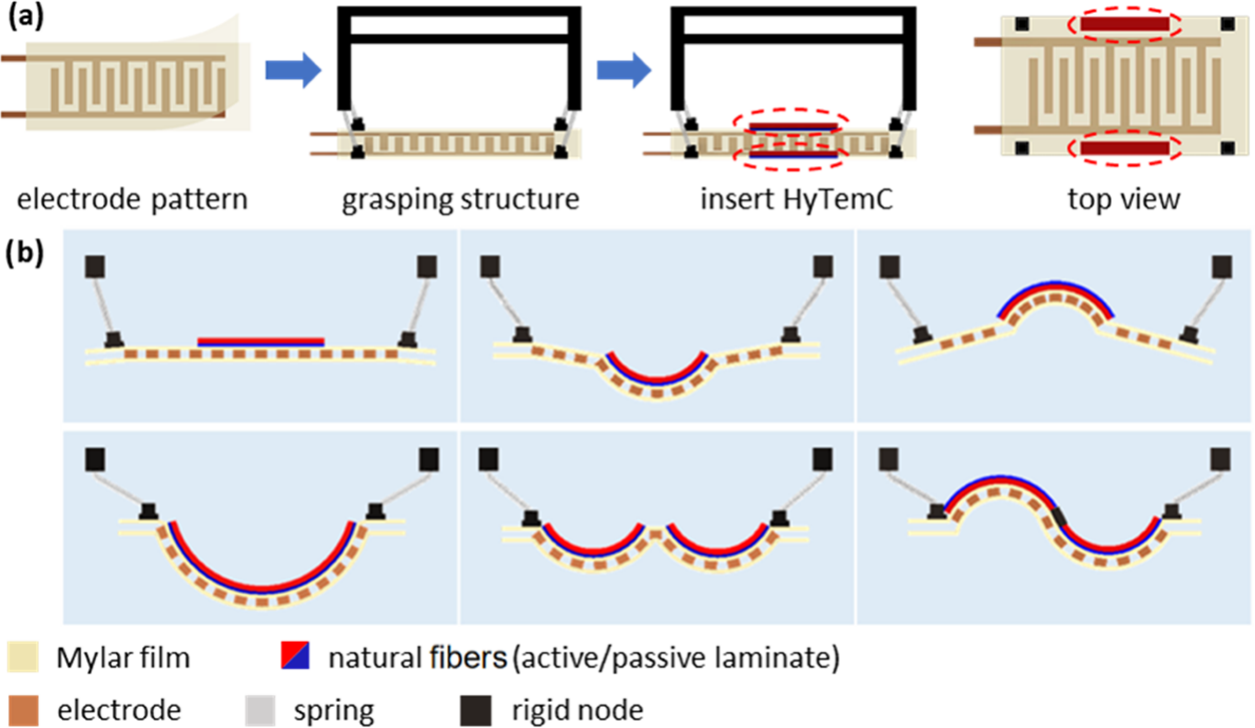
Layout of the design of the whole electroadhesive metasurface with HyTemCs and demonstration of the broad range of possible gripping shapes. (a) Active shape morphing surface is the combination of HyTemC materials and a flexible contact EA film. HyTemCs can be programmed to one shape with different initial moisture contents that allow for the desired autonomous morphing in different operational environments. The motion of the flexible adhesive surface is facilitated by connecting low-stiffness springs to the rigid base. (b) Wide range of gripping shapes is achievable based on different initial moisture contents used during the programming steps and different HyTemC architectures, lengths, and numbers of HyTemCs used in the configuration of the biobased electroadhesive metasurfaces.

The electroadhesive films are produced by stacking a stretchable electrode and an insulated film with size of 180 mm × 100 mm. A stretchable electrode made of copper material with 6.4 mm in width and 0.04 thickness (procured from RS Components, UK) is cut and patterned like in Figure 3a. The electronic circuitry is protected by a transparent polyurethane conformal coating (provided by Electrolube UK) and sealed by insulated Mylar film with 0.023 mm thickness (RS components UK). The substrate base has the exact area size of the electroadhesive surface, with four low stiffness stainless-steel springs (spring constant of 0.16 N/mm, outside diameter 3.52 mm and free length 29.3 mm) at the boundary. The more rigid substrate is necessary because the active morphing surface requires stable movements and the facility to place and connect the metasurface to other devices. The low stiffness springs also provide a flexible and adaptable connection to other supports; stiffer springs or connectors would otherwise constraint the motion of the active morphing part of the metasurface. To maintain the initial flat shape of the adhesive surface, a pretension is provided by the four springs when they are tilted by 10 mm toward the middle section along the longitudinal direction (Figure 3a,b, the first case). The pretensile force is needed to avoid the sagging of the metasurface due to gravity. The programmed HyTemC materials are bonded to the top electroadhesive surface by a double-sided adhesive tape and positioned on the two sides to create an axial curved surface. The actuated shapes vary due to the inserted HyTemC units, all with different initial moisture contents. The programmed actuation depends on the architecture of the biobased hygromorphs units (active layers on top or bottom) and their different lengths and numbers over the adhesive surface. Broad ranges of gripping shapes are shown in Figure 3b.

## 3 Results and Discussion

### 3.1 Design of the HyTemC

**Figure 4:**
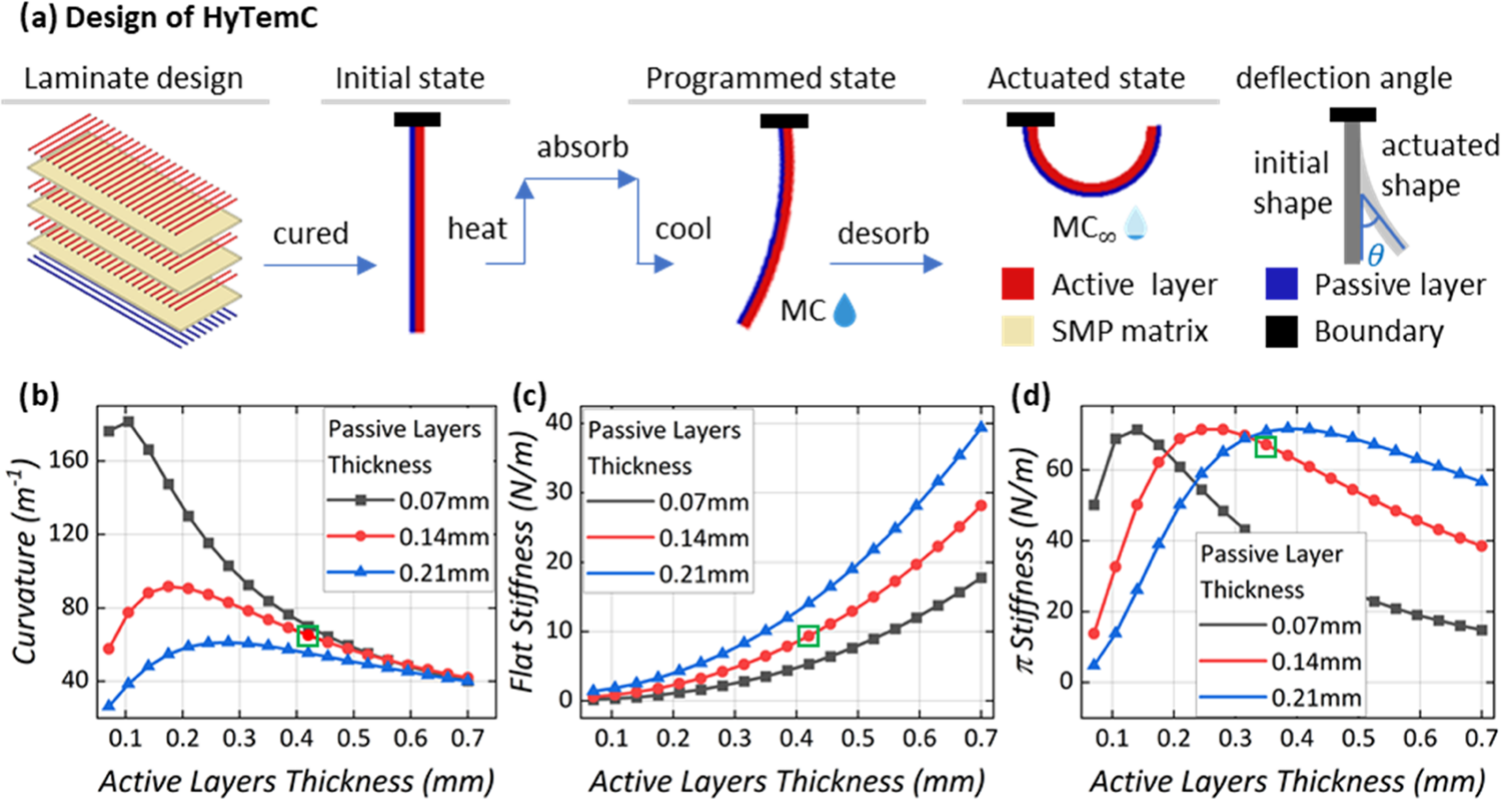
Design of the HyTemCs based on ranges of actuation curvatures and their mechanical properties. (a) HyTemCs with unbalanced architectures consist of active and passive layers of long flax fiber reinforcements embedded within the SMP matrix. Initial flat HyTemC stripes absorb moisture above the *T*_g_ temperature to store predetermined moisture levels but maintain nearly flat shapes during the programming steps. Programmed HyTemCs achieve autonomous bending when exposed to the room environment (50% RH) to reach highly curved shapes. The ranges of the actuated curvatures and the mechanical properties of the HyTemCs are designed by controlling separately the thickness of the active and passive layers. (b) Curvature ranges and (c) bending stiffness at the initial state with flat shapes and (d) at the actuation state with π angle deflection vary with the different thicknesses of the active and passive layers. The green square markers are related to the selected thickness values.

The HyTemCs architectures and stacking sequences are determined based on two specifications: the range of required actuating curvatures and the bending stiffness. Three states of the HyTemCs are possible during use: initial, programming, and actuation. The initial state is related to the flat biobased hygromorph after curing. The programming state corresponds to a configuration with a small level of bent shape, which is related to the presence of internal moisture content. The actuation state involves highly bent shapes that result from desorbing all of the predetermined moisture contents—see Figure 4a. The calculated curvature ranges are from programming to actuation states, with moisture contents from immersion in water to 50% relative humidity. The prediction of the longitudinal curvature variation Δ*K* is based on the use of modified Timoshenko equations (eqs 1 and 2). The results related to the curvature ranges are shown in Figure 4b. The selected values of the overall passive layer thickness are because of the average thickness of one lamina (0.07 mm). If the passive layer thickness is maintained constant, the curvature ranges increase sharply to a maximum value and then decrease as the active layers become thicker. The maximum curvature for each passive layer value decreases as the passive layers’ thicknesses become larger. The green square in Figure 4b is related to the values of the thickness for the active and passive layers chosen for the design of the biobased hygromorph electroadhesive metasurfaces, with a resulting curvature of 65.08 m^–1^; this value is deemed sufficient to cover the multiple shapes that the metasurface must handle.
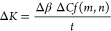
1
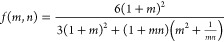
2In eq 1, Δβ is the difference in the coefficients of hygroscopic expansion (CHE) between active and passive layers, Δ*C* is the water loss between wet and dry states, and *t* is the total thickness of the hygromorph. The terms  and  have *t*_p_ and *t*_a_, which represent the passive and the active layer thicknesses. *E*_p_ and *E*_a_ are the tensile modulus of the wet passive and active layers, respectively.

The stiffness of the hygromorph is here evaluated from the initial to the actuation states (Figure 4c,d, respectively). The stiffness is calculated using a previously discussed approach.^22^ The shapes of the samples at the initial state are flat, and their corresponding stiffness has a positive correlation to the thickness of the active and passive layers. A higher content of passive layers also improves the stiffness of the flat hygromorphs when the overall thickness is constant because the passive layers have at least 5 times larger tensile modulus than the active ones (see the Supporting Information). The shapes of different laminates during the actuation state are different because of their dissimilar curvature ranges. A comparative configuration is the one with the same deflection angle (θ) π, which means that stripes with lower curvature ranges require larger lengths to reach the same deflection angle (θ). The selected laminate designs are indicated by the green squares in Figure 4, and take into consideration operational curvature ranges, i.e., the stiffness of the flat and π deflected hygromorphs. The final thickness values of the active and passive layers are 0.42 and 0.14 mm, respectively.

### 3.2 Design of the Electroadhesion Pattern

**Figure 5:**
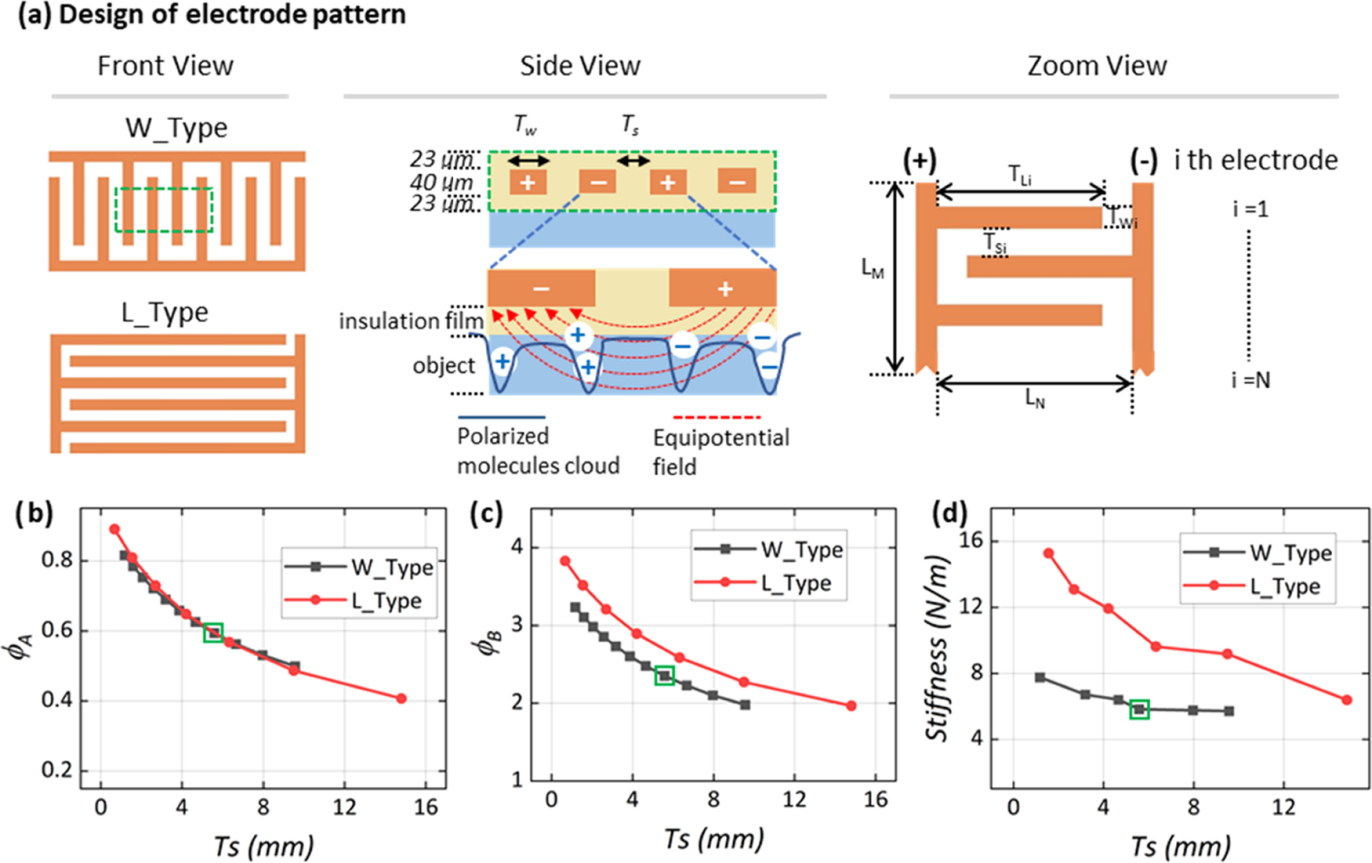
Design of the electrode pattern for the biobased metasurface. The design is based on the adhesive force and the bending stiffness. (a) Electrode pattern is designed for different configurations (W_type or L_type), electrode lengths, and widths *T*_L_ and *T*_w_, and the space between two electrodes *T*_s_. Equipotential field distributions generated by the two in-plane electrodes propagating in the out-of-plane direction provide the maximum charges accumulated in the areas of the object corresponding to the boundary edges of the electrodes so that higher ϕ_A_ and ϕ_B_ fractions help to achieve larger adhesive forces; their relationship with *T*_s_ is shown in panels (b) and (c). Lower bending stiffness provides less resistance when the biobased HyTemC is actuated. (d) W_type configuration and wider space between electrodes help to lower the bending stiffness. The selected type of electrode positioning and *T*_s_ value for the final design are indicated by the green squares.

The electrode layer should possess a low bending stiffness for the HyTemC to actuate. To maximize the surface bonding, the electroadhesion layer must generate high adhesive forces at a constant voltage. The geometry parameters of the electroadhesive film are shown in Figure 5a. The patterned electrode can be aligned along the length (L_Type) or width (W_Type) directions. The adhesive film has a three-layer sandwich structure (insulated Mylar film/electrode/insulated Mylar film), with thicknesses equal to 23/40/23 μm, respectively. The length and width of the electrodes are marked as *T*_L_ and *T*_w_ (6.4 mm), and the space between the two electrodes is marked as *T*_s_. The available perimeter length (*L*_M_) and width (*L*_N_) for the electrode are 150 and 70 mm, with an edge smaller than the overall size 180 mm × 100 mm of the whole surface to host the HyTemC actuators and the spring connectors. We define here the electrode areal fraction (ϕ_A_) as the ratio between the electrode surface and the electroadhesive-device areas (150 mm × 70 mm). The boundary-edge ratio ϕ_B_ is defined as the sum of the boundary-edge lengths divided by the perimeter of the device size
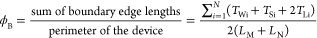
3The results of ϕ_A_ and ϕ_B_ versus *T*_s_ are shown in Figure 5b,c. When *T*_w_ is constant (6.4 mm in our case), the fractions ϕ_A_ and ϕ_B_ are positively related to the surface adhesion force.^3^ The fraction ϕ_A_ is not sensitive to the direction of the electrode distribution (L_type and W_type). In the case of ϕ_B_, however, the L_type shows a better performance than the W_type. Narrow spaces between electrodes help to reach higher values of both ϕ_A_ and ϕ_B_. To obtain higher electroadhesive forces, an efficient approach is to use photolithography technology with slim electrodes and narrow spaces between them;^3^ however, this is not a focus of our work. The stiffness of the electrode sandwich structure is analyzed via Figure 5d using finite element simulations. The ideal electrode stiffness would be as small as possible for the structure to bend to highly curved shapes. The stiffnesses of the L_type electrode configurations are significantly larger than those of the W_type because the L_type copper electrode is continuous along the longitudinal direction but discontinuous in the case of the W_type. Values of *T*_w_ = 6.4 mm and *T*_s_ = 5.6 mm were selected, and a W_type electrode was used in subsequent experiments, as marked by the green squares in Figure 5b–d.

### 3.3 Biobased Electroadhesive Metasurface in Operation

**Figure 6:**
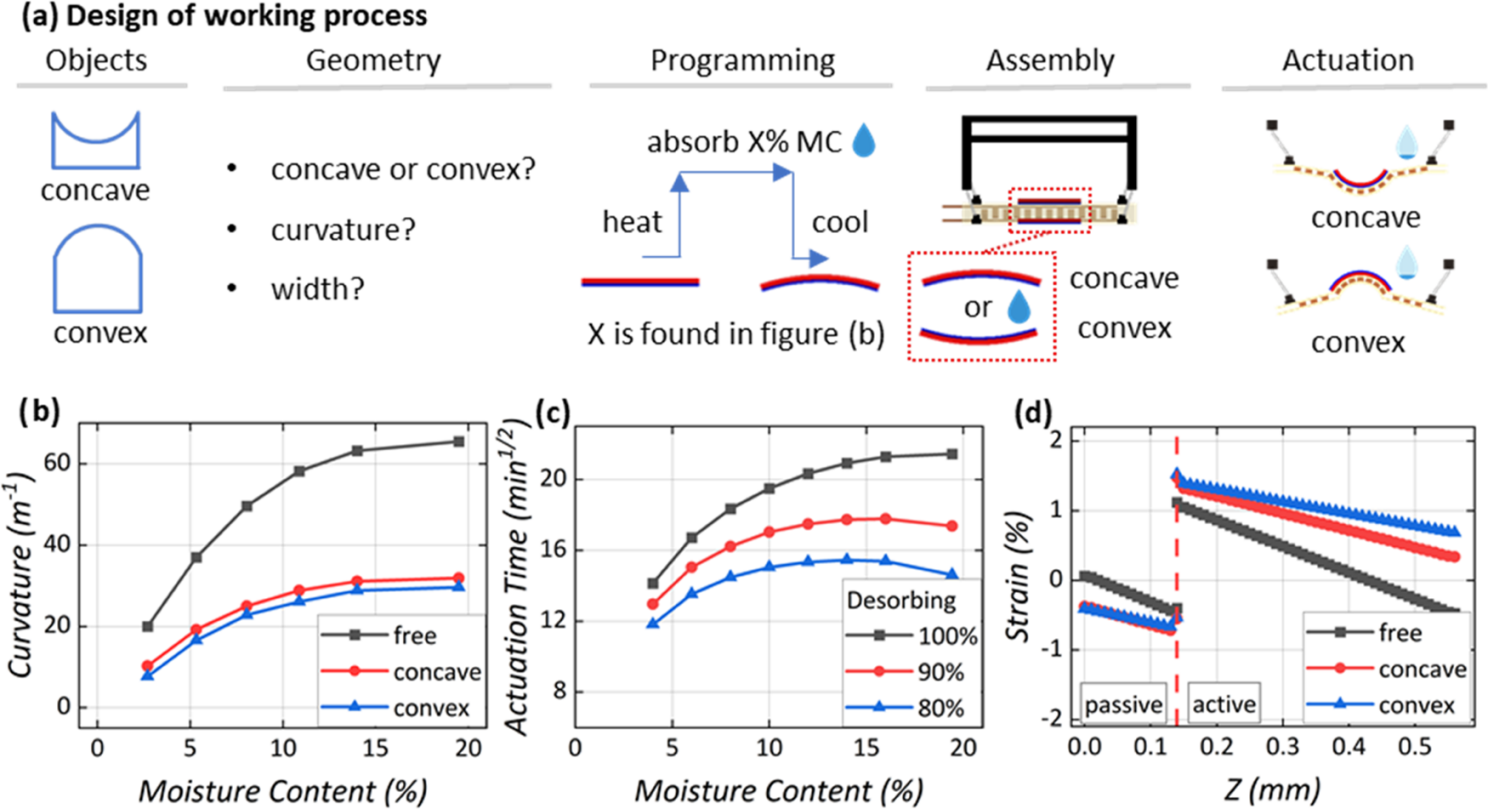
(a) Objects to be grasped have a broad range of shapes and sizes. The geometry characteristics of the objects are collected as input parameters for the programming steps. Strips store *X*% of moisture and maintain nearly flat shapes during programming and are then assembled into the metasurface. The HyTemCs will desorb *X*% moisture autonomously when exposed to a room environment (50% RH, as an example), reaching the predefined actuated shapes. The *X*% moisture for the designed shapes is found in panel (b). The actuation time varies with the desorption—see panel (c). (d) Strain distributions for the HyTemCs in the three conditions (free, concave, and convex) through thickness from passive to active layers. The results are obtained from finite element models.

The principle of operation of the biobased electroadhesive metasurface is shown in Figure 6a. The input geometry parameters are derived from the shapes of the target objects (concave, convex, or multiple shapes), their curvatures, and widths. The programming and assembly steps of the HyTemCs are based on the geometry parameters described above. The designed curvatures are controlled by the initial moisture content, and those correlations are shown in Figure 6b. The HyTemCs are stored at a temperature above *T*_g_ while absorbing the initial moisture until reaching the corresponding weight values. The programmed and cooled HyTemCs are placed onto the target surface. The orientation of the positioning (active layers on top or at the bottom) controls the type of actuated shape. If the active layers are on the top, the morphed shapes are concave, the opposite otherwise. The curvatures provided by the metasurface with free boundary joints only are significantly higher than those when the EA grippers are placed (Figure 6b). Curvatures produced by the metasurface via concave or convex configurations differ very little (see also Figure 6b). The assembled metasurfaces are here placed at room condition (20 °C and 50%, in our example). The shape morphing happens autonomously because the initial moisture desorbs at room environment to reach the predefined shapes. Desorption times to stability related to different initial moisture contents without any moisture diffusion are shown in Figure 6c. The effect of partial desorption (90 and 80%) is also shown in Figure 6c; the final 10–20% of moisture loss takes significantly more time than the same amount of moisture loss at the beginning. The final 10–20% of moisture diffusion also contributes less to the deformation but entails a significant portion of time, so the most efficient method to improve the actuation time versus the moisture content is at 80% relative desorption (blue line in Figure 6c). The strain distributions along the thickness of the HyTemCs (from passive to active layers) are shown in Figure 6d. The bending strains within the HyTemCs under free boundary conditions are slightly lower than those related to the concave and convex conditions.

**Figure 7:**
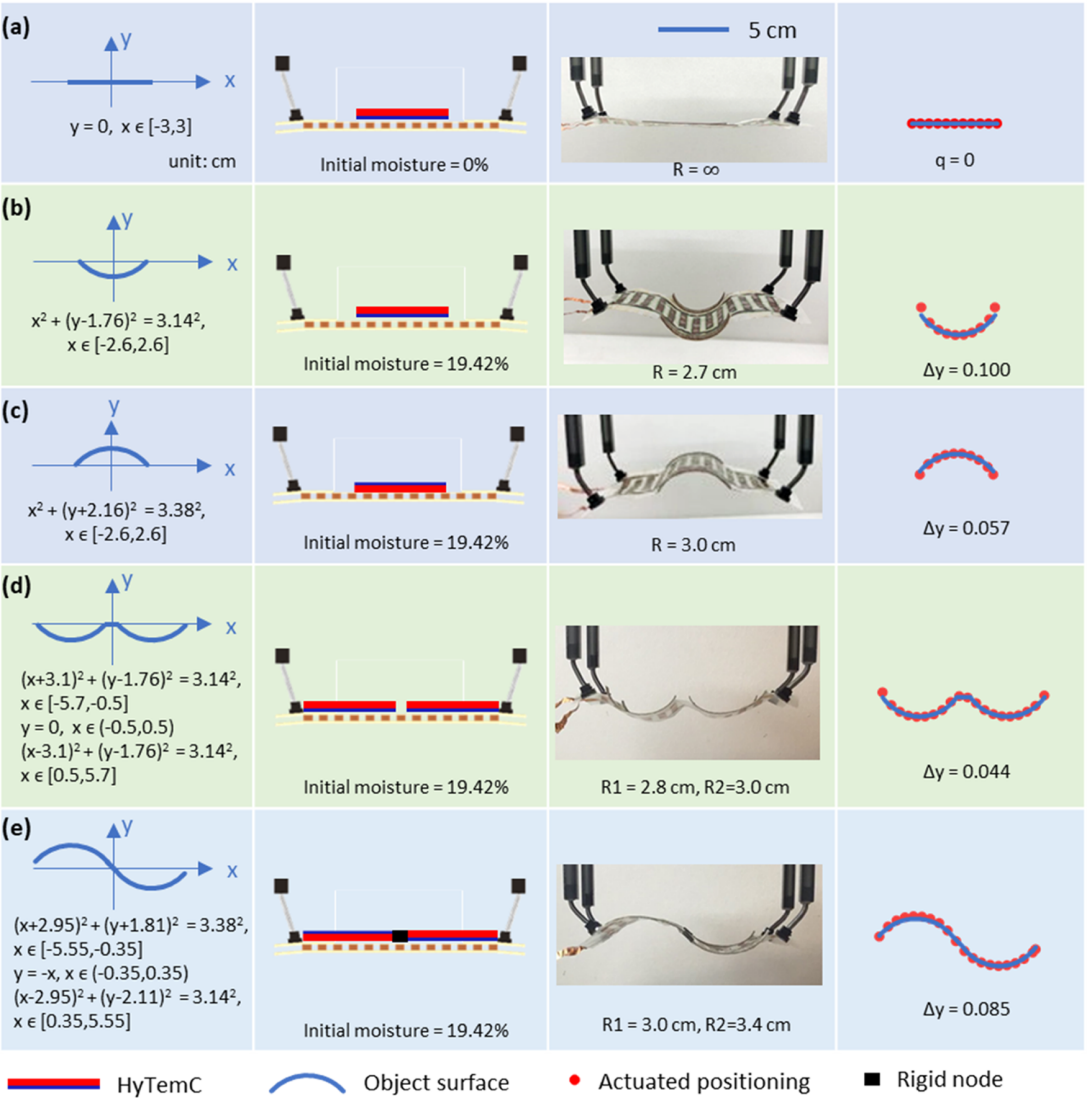
Five cases representing a wide variety of possible shapes that the biobased metasurface can reproduce and adapt to (a) flat, (b) concave, (c) convex, (d) dual concave, and (e) wavy shapes. The object surface functions are displayed in the first column of the table. The initial moisture content during the programming steps and the assembly forms are shown in column two. The experimental curvatures (third column) show some slight differences from the design target. The experimental actuated positions are marked in red points (column four), and the differences with the target object surface (blue lines) are calculated as Δ*y*.

Examples of the shapes that the biobased metasurface can provide are shown in Figure 7. Those shapes are representative of just five examples, and more shapes can be easily obtained by controlling the different initial moisture contents, fiber directions, length, and a number of units. The five examples include (but are not limited to) flat, concave, convex, dual concave, and convex–concave shapes. The maximum initial moisture content is at 19.4%; this means that the examples of Figure 7b–e are those that possess the largest curvature. The values of the radius obtained from the flat configuration are between 2.7 and 3.4 cm, depending on the different boundaries. The error between the theoretical values is shown in Figure 4b, and the measured curvatures are shown in the last column (red points are indicative of the experimental positions, and the blue lines are the target design curves). The linear error Δ*y* is calculated using the Euclidean norm—see eq 4.
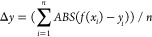
4In eq 4, *f*(*x*_*i*_) are the vertical (out-of-plane) values of the target design surfaces, and *y*_*i*_ represents the measured positions after actuation.

Videos of Figure 7d,e are included in Video S1 and have been captured at 1080P HD, 30 fps.

**Figure 8:**
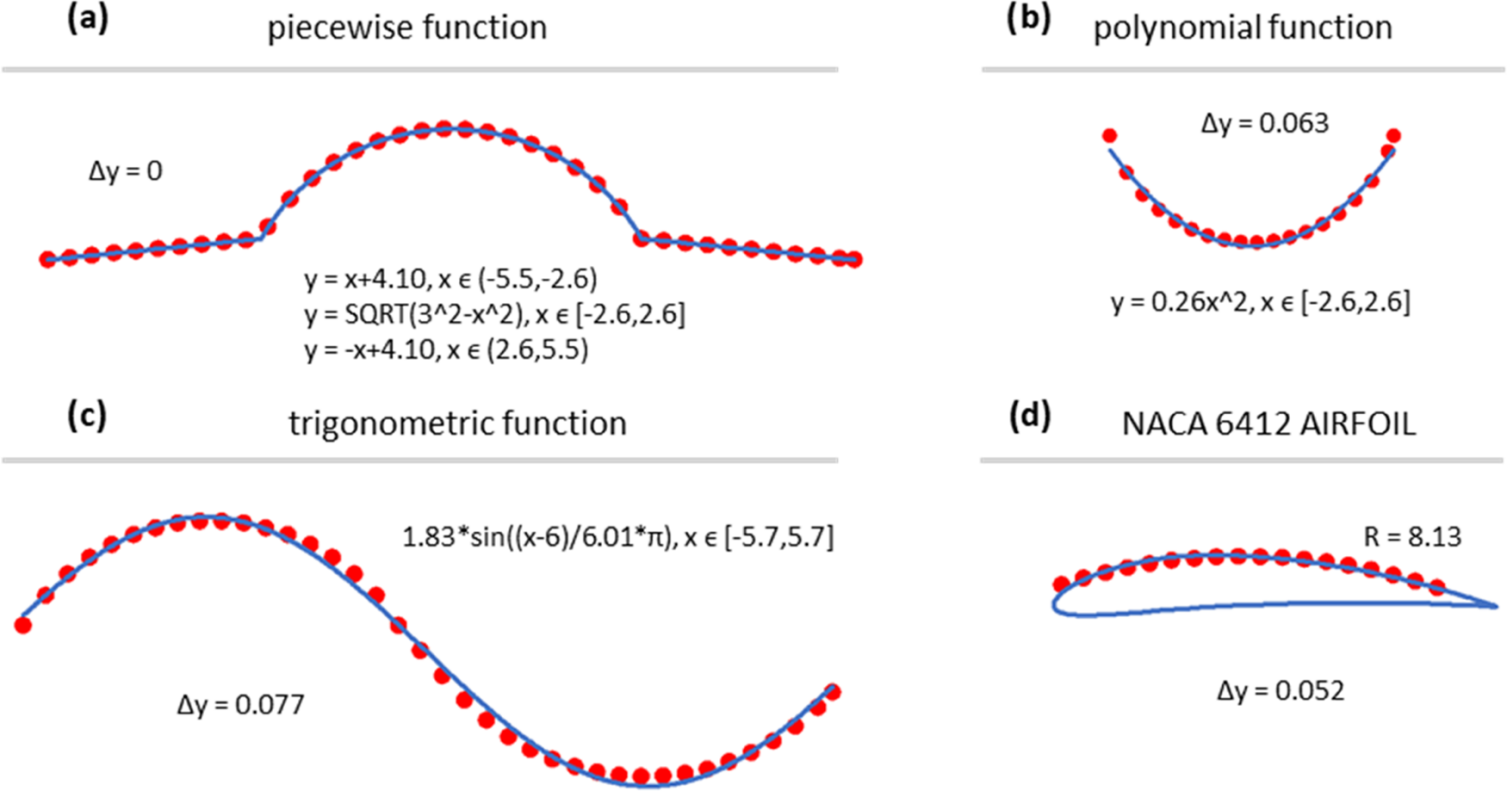
Example surface profiles that can be adopted are not restricted to circular shapes but also extended to other functions such as (a) piecewise, (b) polynomial, (c) trigonometric, and even (d) a NACA 6412 airfoil example. The red points in panels (a)–(d) are the experimental structure coordinates, while the blue lines represent the object surfaces.

In addition to the above-mentioned circular curves, a wide variety of other forms can also be constructed as target actuated curves, such as piecewise, polynomial, and trigonometric. An example of a more complex curve is the NACA 6412 airfoil (see Figure 8). These higher-order actuated shapes are well reproduced by our biobased electroadhesive metasurface.

## 4 Conclusions and Future Work

The biobased and programmable environmentally stimulated electroadhesive metasurfaces described in this work have the following significant features:

(a) Capacity to be programmed and to adapt to multiple and complex object shapes (flat, concave, convex, dual concave, and wavy shapes (Figure 7)). Good agreement is observed between the experimental and the target functions representing the objects, even when the shapes are complex.
(b) No external contact forces are required. The active and autonomous shape-changing performances provided by the biobased HyTemCs used in the metasurface allow a high-precision match between the actuated surfaces and the objects to be handled. Only the electroadhesive force is applied to the objects to overcome gravity, with negligible compressive forces. This feature makes the proposed biobased metasurface highly suited to handle fragile objects.
(c) Development of large curvatures after actuation. Actuator curvature can reach a maximum of 31.89 m^–1^ for concave bending and 29.63 m^–1^ for convex flexural deformation. These values can cater for a very wide ranges of curvatures, from flat to highly curved shapes.
(d) Autonomous morphing. The actuation process is autonomous and triggered by the operational environment only. This avoids the use of large, heavy, and energy-intensive actuation devices.
(e) Variable stiffness. The biobased HyTemC is relatively soft (6.6 GPa) in an immersed state but rigid (13.8 GPa) during actuation—see Figure S1e,f. This prevents the onset of peeling, especially when the HyTemC is required to lift objects with weights.
(f) Biobased materials used for flexible and sustainable robotics. The hygromorph HyTemCs are made of natural fibers (flax, in our case), which are sustainable and environmentally friendly. The use of the HyTemCs within the metasurface concept can contribute to developing smart structures and robotics applications that lower their carbon and general emissions footprint.

The drawback is represented by the actuation time of the devices made with our HyTemCs (approx 3 h, see Figure 6c). The increase of fiber volume fraction and thinner samples would improve the speeds of moisture absorption/desorption in future works. Also, the gripper requires new metasurfaces to adapt to new surfaces, and the whole efficiency of the system would be enhanced by programming and actuating the metasurface units with local thermal and humidity fields. The actuator unit is an environmentally friendly material, and the gripper structure will be biodegradable or recyclable if all elements are from natural resources.

## 5 Experimental Section

### 5.1 Moisture Content Characterization

Manufactured and cut samples were stored in a reference state in a Votsch climatic chamber, which controlled the RH at 50% and the temperature at 23 °C. The samples were weighed using a balance with 10–3 g precision (PNS 600-3 Kern, Germany). Moisture contents at various RHs (Figure S1a) and immersion over time (Figure S1b) were characterized
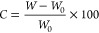
5where *W* and *W*_0_ are the weight of the sample at various RHs or immersion over time and the weight of the dry material before immersion (for RH = 50% and *T* = 23 °C).

### 5.2 Expansion Measurement

The hygroscopic expansion and moisture uptake have been evaluated on samples with dimensions 70 mm (*L*) × 10 mm (*w*) × 0.56 mm (*t*). Geometry measurements have been performed with a Mitutoyo micrometer IP65. Gravimetric analyses have been carried out using a balance of 10^–3^ g precision (PNS 600-3 Kern, Germany). The coefficient of hygroscopic expansion (β) has been determined as the slope of the hygroscopic expansion over the moisture content. The results are shown in Figure S1c.

### 5.3 Elastic Properties

The tensile properties of dry and wet unidirectional biomaterial composites (no EAs) with flax-fiber orientations set at 0° (*E*_L_) and 90° (*E*_T_) have been measured according to ISO 527-4 standards using a Shimadzu universal testing machine (cell load 5 kN) at controlled temperature with a crosshead speed of 1 mm/min. The samples have the following dimensions (thickness *t* and width *w*): *t*_0°_ = 0.56 mm and width *w*_0°_ = 15 mm; *t*_90°_ = 0.56 mm and *w*_90°_ = 25 mm. Mechanical tests were performed on samples that had reached their saturation time. The samples were covered with a polyethylene wrap to prevent the loss of moisture during the tensile process. A heating chamber (TCE-N300A, Shimadzu, U.K.) controlled the setting temperature, while thermocouples have also been used to verify the temperature close to the samples. The tensile modulus was determined within a range of strains between 0.05 and 0.1%. The results are shown in Figure S1e,f.

### 5.4 Measurement of the Curvature

To measure the radius of curvature, markers were tracked on images captured using a camera (1080P HD, 30 fps). The image data processing analysis was performed using Autodesk software. The curvature was measured by fitting the sample profile to a “circle” function. The bending curvature (*K*) was calculated from the radius of the fitted circle.

## Supporting Information Available

Experiments related to the HyTemC hygromorph material for the units of metasurface; moisture conditions at various relative humidity (RHs) for different laminates (Figure S1a); moisture diffusion from 50% RH to immersion (Figure S1b); evolution of the longitudinal and transverse hygroscopic strains at 20 and 100 °C (Figure S1c); DMA experimental results related to the pure epoxy resin, showing a *T*_g_ close to 60 °C (Figure S1d); evolution of the transverse and longitudinal Young’s moduli as a function of temperature (Figure S1e); and evolution of the transverse and longitudinal Young’s moduli as a function of moisture content (Figure S1f); initial moisture content programmed in various ways within the composite (Figure S2); curvature ranges for three different laminates after 10 cycles from RH 50% to immersed states (Figure S3a); curvature changes during the programming and actuating processes after 10 cycles (Figure S3b); (PDF)
Video of biobased and programmable metasurface for ultracomplex shape electroadhesive robotics handling (Video S1) (MP4)
