# Supplementary material for: Biobased and Programmable Electroadhesive Metasurfaces
Source: ACS Appl Mater Interfaces. 2022 Oct 6;14(41):47198–208. doi: 10.1021/acsami.2c10392 (PMC9585522; doi:10.1021/acsami.2c10392)
Supplement: Supplementary file 2 — am2c10392_si_002.pdf [file am2c10392_si_002.pdf]

Supporting information

Biobased and programmable electro-adhesive metasurfaces

Qinyu Li[a], Antoine Le Duigou[b], Jianglong Guo[c], Vijay Kumar Thakur[d,e], Jonathan Rossiter[f], Liwu Liu[g], Jinsong Leng[h] and Fabrizio Scarpa[a]**

[a]Bristol Composites Institute, University of Bristol, BS8 1TR Bristol, UK.

[b]Polymer and Composites, Université Bretagne Sud, IRDL UMR CNRS 6027, F-56100 Lorient, France

[c]School of Science, Harbin Institute of Technology (Shenzhen), Shenzhen, 518055 People's Republic of China

[d]Biorefining and Advanced Materials Research Center, Scotland's Rural College (SRUC), Kings Buildings, West Mains Road, Edinburgh, EH9 3JG UK

[e]School of Engineering, University of Petroleum and Energy Studies (UPES), Dehradun, 248007 Uttarakhand, India

[f]SoftLab, Bristol Robotics Laboratory, University of Bristol, Ada Lovelace Building, University Walk, BS8 1TW Bristol, UK

[g]Department of Astronautical Science and Mechanics, Harbin Institute of Technology (HIT), P.O. Box 301, No. 92 West Dazhi Street, Harbin 150001, P. R. China

[h]National Key Laboratory of Science and Technology On Advanced Composites in Special Environments, Harbin Institute of Technology (HIT), No.2 Yikuang Street, P.O. Box 3011, Harbin 150080, P. R. China.

[a*]E-mail: F.Scarpa@bristol.ac.uk

[g*]E-mail: lengjs@hit.edu.cn

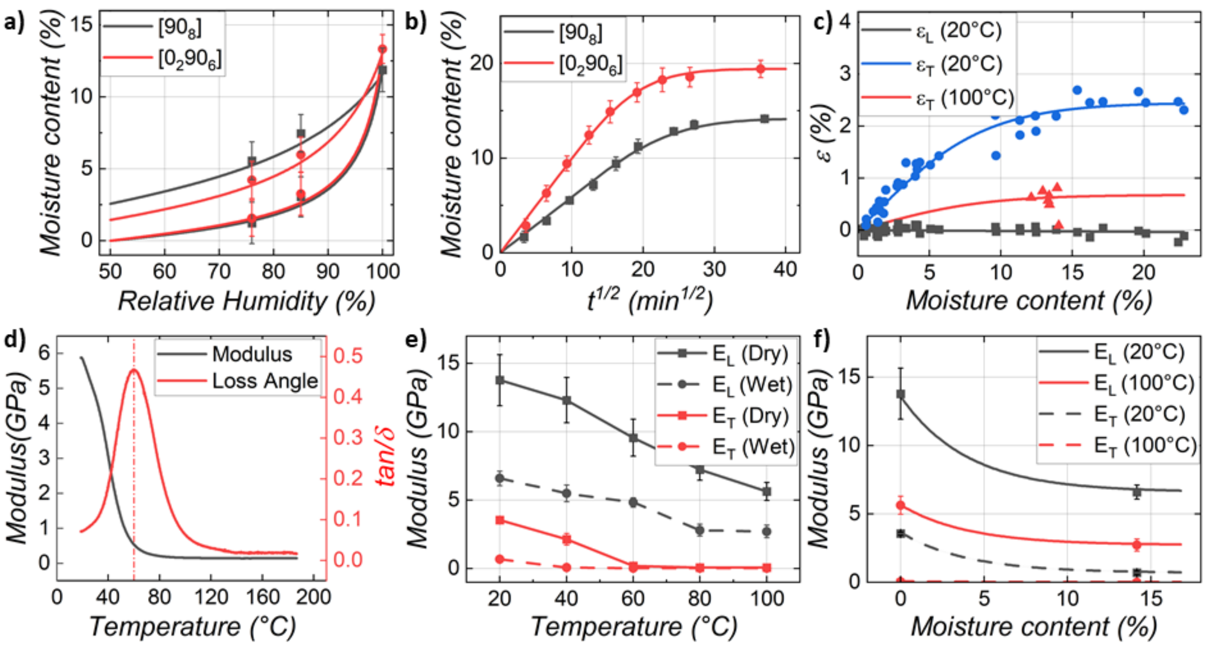

Figure S1 Experiments related to the HyTemC material: a, b) Diffusion kinetics of the unidirectional [90_8] and asymmetrical [$0_2 90_6$] biocomposites. a) Moisture conditions at various RHs for different laminates. Slightly differences between absorption and desorption are due to hysteresis. b) Moisture diffusion from 50% RH to immersion. The variation of moisture content is very small within the different actuators. c) Evolution of the longitudinal and transverse hygroscopic strains at 20 °C and 100 °C. Red, black and blue dots represent the experimental data and the continuous refer to the theoretical results from the model. d) DMA experiment results related to the pure epoxy resin, showing a Tg around 60 °C. e) Evolution of the transverse and longitudinal Young's moduli as a function of temperature. Dry and wet states are corresponding to 50% RH and immersed respectively. f) Evolution of the transverse and longitudinal Young's moduli as a function of moisture content.

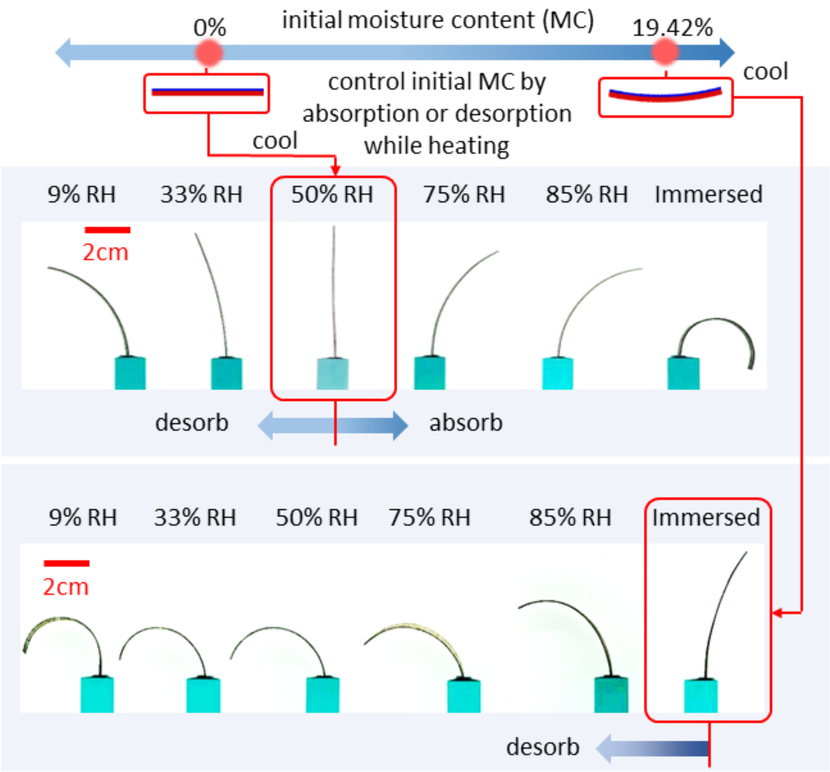

Figure S2 The initial moisture content can be programmed in various ways within the composite. Every defined initial MC level creates new sets of shape at different humidity conditions. Two examples with their shape sets are here illustrated for initial MCs of 0% (50% RH) and 19.42% (immersed). The programming allows for a multifunctional shape transformation: shapes can be initially designed for any humidity condition, the autonomous actuation is stimulated by the environmental surrounding only. Reversible and repeatable morphing is achieved with locking the shape at constant humidity.

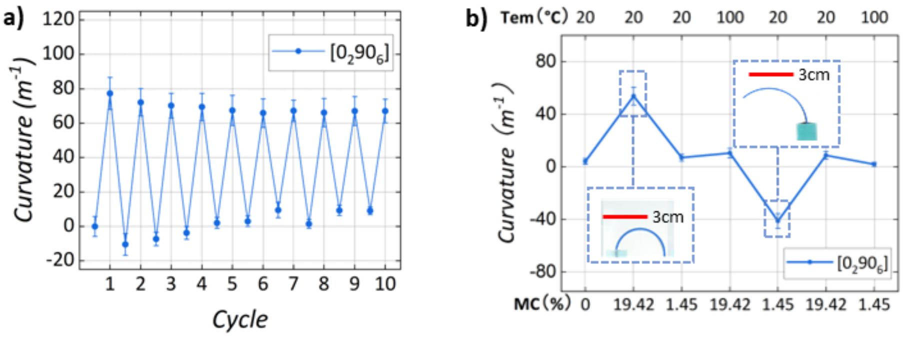

Figure S3 a) Curvature ranges for three different laminates after 10 cycles from RH 50% to immersed states. b) Curvature changes during the programming and actuating processes after 10 cycles.

Modeling the actuation of the electro-adhesive metasurfaces

The Finite Element model of electro-adhesive metasurfaces is representative of the structural stiffness of the system for the electrode pattern design shown in Figure 5d) and the actuation curvature, actuation time and evaluation of the internal stresses in Figures 6b) to d).

Stiffness evaluation by designing the eletrode pattern

The overall structure has three parts: a mylar film/electrode pattern/mylar film, with thicknesses equal to 23μm/40μm/23μm. The electrode pattern is controlled by changing the distribution type (W_type or L_type) and Ts, the latter being the space between two electrodes. The materials properties and their types of elements and mesh size are shown in **Table S1.** The analysis is Static nonlinear slover in Abaqus 6.14. One boundary is fixed to one side of the mylar film/electrode pattern/mylar film structure, and a concentrated force of 0.01N is applied to the other side. The ratio between the applied force and the corresponding displacement provides the equivalent bending stiffness of the structure.

Prediction of the actuation curvatures and related times, and the evalution of the internal stresses

The whole biobased metasurface actuator structure contains the mylar film/electrode pattern/mylar film, the inserted HyTemC, springs and a substrate of Polylactic acid (PLA), the latter made via 3D printing. The assemly is shown in Figures 3 and 6. The properties of the HyTemC inserts are from Figure S1c) for the hygroscopic expansion, and Figure S1f) shows the elastic properties with different moisture contents. Properties of the other materials and their types of elements and mesh

size are in Table S1. The analysis is Static nonlinear slover in Abaqus 6.14. There is no extra boundary condition applied, except for the moisture field. The loading is the one designed at the initial mositure content to desorb during operational conditions. The actuated curvatures of the free boundary of the HyTemC (concave and convex conditions) are measured by calculating the coordinates of three points (Figure 6b)) and the desorbing time (Figure 6c)). The internal stresses through the thinkness in the HyTemC at free, concave and convex configurations are extracted from the Finite Element model and averaged (see Figure 6d)).

Table S1 Materials properties and element setting for the modelling

	Density (g/cm^3)	Elastic property (MPa)	Element size (mm)	Element type
Electrode (copper)	8.96	117000	0.5*0.01(plane*thickness)	C3D8R
Mylar film	1.38	2800	1*0.01(plane*thickness)	C3D8
Base (PLA)	1.25	4000	3	C3D10
Spring (steel)	8.00	300000	0.15	C3D4
